# Bardet-Biedl Syndrome with a Kidney Transplant, Esophageal Adenocarcinoma, and Postoperative Complications

**DOI:** 10.1155/2019/8983174

**Published:** 2019-07-02

**Authors:** Georgi Yankov, Teodora Mihalova, Rosen Petkov, Evgeni Mekov, Stefka Yankova, Danail Petrov

**Affiliations:** ^1^Department of Pulmonary Diseases, Medical Faculty, Medical University-Sofia, MHATPD “Sveta Sofia, ” 19, “Acad. Ivan Geshov” Blvd, Bulgaria; ^2^Department of Nephrology and Hemodialysis “Prof. I. Mitеv, ” Medical Faculty, Medical University-Sofia, 11, “Acad. Ivan Geshov” Blvd, Bulgaria

## Abstract

The authors present a rare clinical case of a patient with Bardet-Biedl syndrome and chronic kidney disease, who reached end-stage renal disease (ESRD) and underwent a long-term hemodialysis treatment, during which infections with Hepatitis C Virus (HCV) infection and Cytomegalovirus (CMV) infection were established. Kidney transplantation from an alive unrelated donor was performed. Later, an adenocarcinoma of the esophagus was diagnosed at an early stage, treated surgically with resection of the esophagus and gastroesophagoplasty afterward. Seven months later, a rare complication of the immunosuppressive therapy with Cyclosporin A occurred, which consisted of spontaneous bilateral pleural hemorrhage. The same, as well as the postoperative ventral hernia, was successfully resolved. Concomitant HCV was also treated. Rare autosomal recessive syndrome with severe complications, adenocarcinoma of the esophagus, spontaneous bilateral pleural hemorrhage after the operation, and successful treatment were discussed.

## 1. Case Presentation

**Figure 1:**
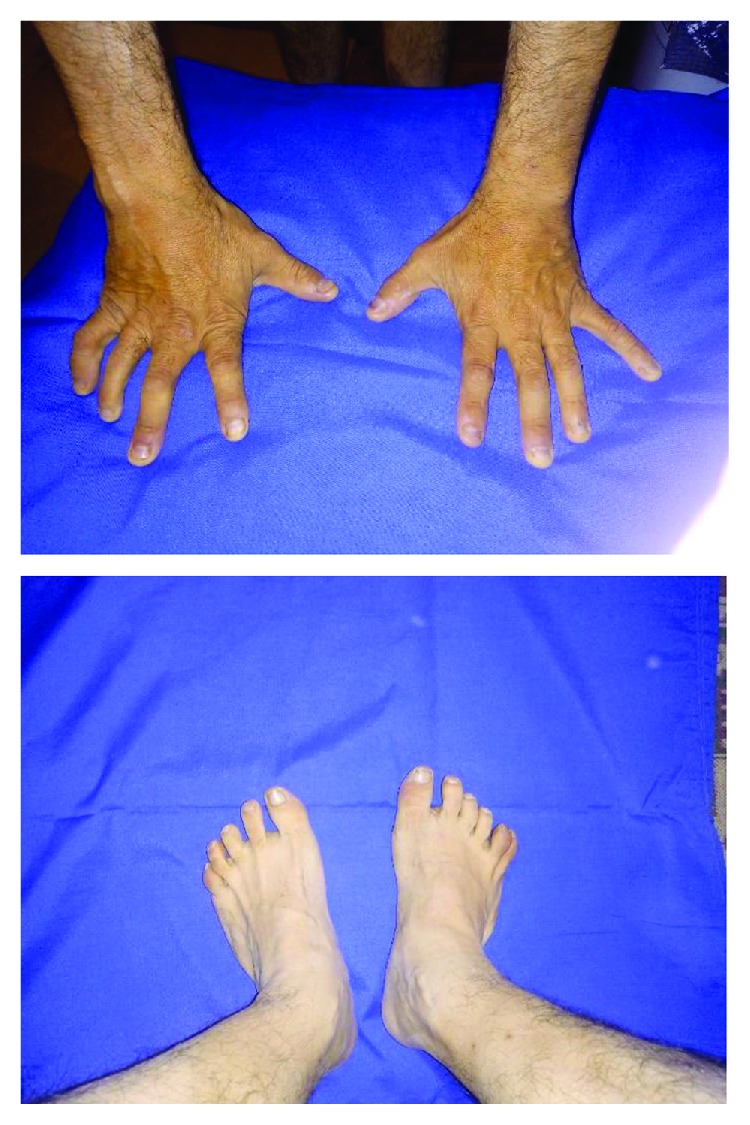
Patients' extremities.

A 33-year-old man was hospitalized at the Thoracic Surgery Clinic (TSC) with complaints dating from about 2 to 3 months, resulting in pain behind the sternum, difficulty in swallowing solid food, and body weight reduction of about 8 kg. The physical exam is remarkable for polydactyly with syndactyly (Figure 1) and obesity. The patient had six digits on both feet and six digits on the right hand. The accessory finger on the left foot was surgically removed. A right orchiectomy was performed at 17 years due to cryptorchidism for cancer prevention. He also reports myopia since he was a child.

Past and concomitant diseases: Bardet-Biedl syndrome, with end-stage renal disease (ESRD) reached and chronic hemodialysis treatment (CHT), during which infection with HCV and secondary arterial hypertension, were established. Kidney transplantation from an alive unrelated donor in Pakistan was performed. An outpatient CMV infection was found, followed by several hospitalizations for active treatment and follow-up. In the family history, there was also a brother with Bardet-Biedl syndrome, who died 9 years after kidney transplantation.

The Computer Tomography Scan (CT) and abdominal ultrasound did not show any pathological result. The fiber esophagogastroscopy visualized a normal lumen and intact mucosa of the esophagus, but at about 30 cm from the dentition in the back part of the wall, an ulcer with a size of 10 mm was seen, with a shallow bottom and rough and eroded thick edges, from which 3 biopsies were taken. The histopathological examination of the biopsies established a low-differentiated adenocarcinoma.

**Figure 2:**
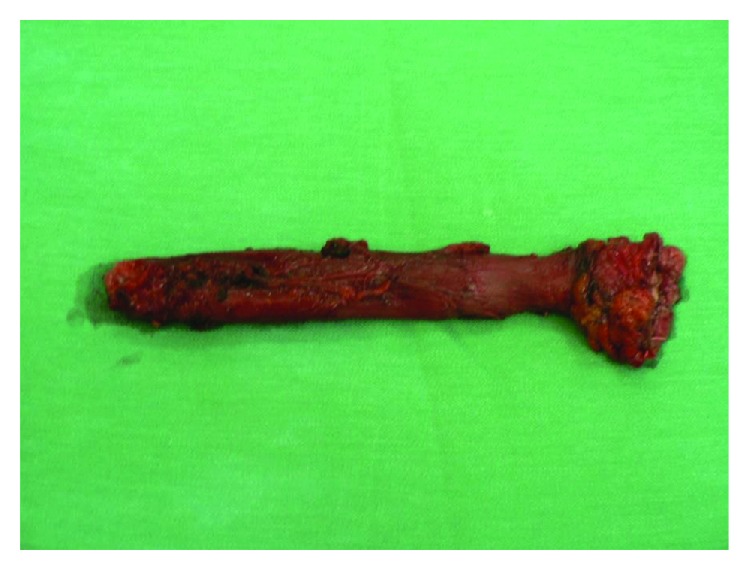
A macroscopic preparation of the resected esophagus.

**Figure 3:**
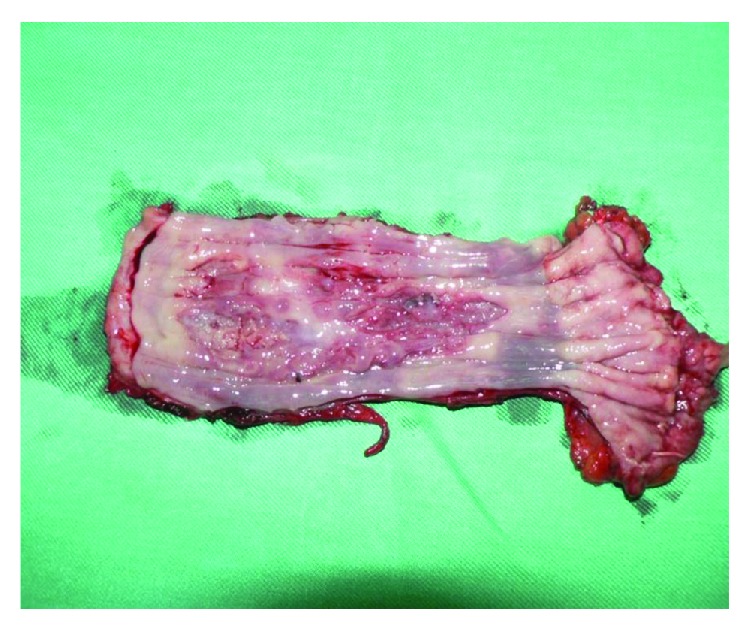
Section of the macroscopic preparation with ulcerative changes.

A surgery treatment was undertaken: subtotal resection of the esophagus, with gastroesophagoplasty *a modo Ivor Lewis–МcKeown* and pyloroplasty with the *Heineke–Mikulicz* method which were performed. On the posterior wall, along with the thoracic esophagus, a discrete seal near 6 cm in length was palpated (Figure 2). During the section of the macroscopic preparation, ulcerative changes with a length of about 6 cm were visualized (Figure 3), the same with definitive histological preparation showing a moderately differentiated adenocarcinoma. In all extirpated lymph nodes, any neoplastic elements were found—there were only nodes with chronic nonspecific lymphadenitis. The patient was released in a clinically stable condition, with a noncomplicated postoperative period following.

**Figure 4:**
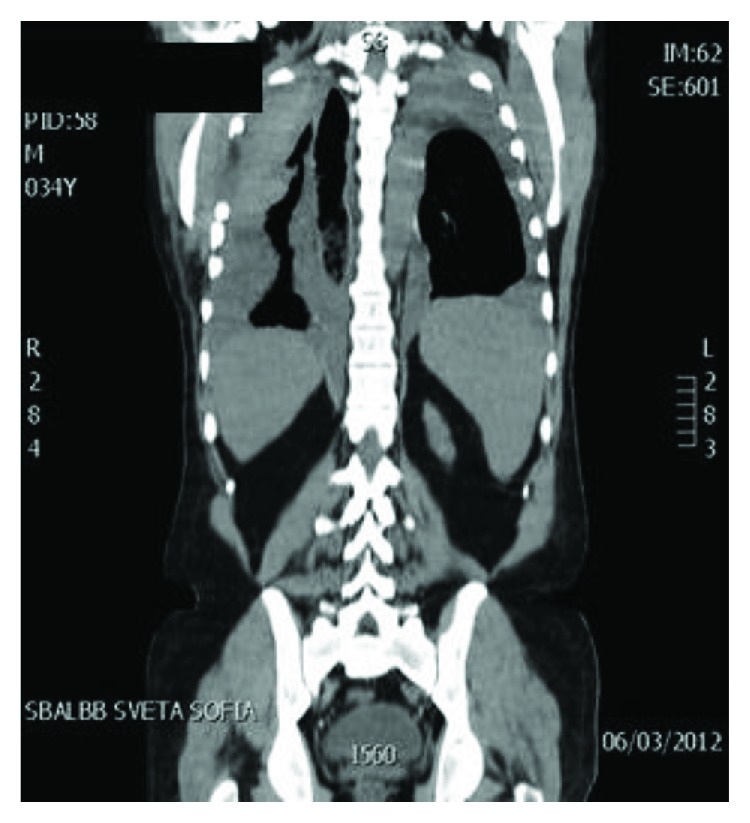
Chest CT—a massive pleural effusion on the right side and a small one on the left.

Seven months later, the patient was hospitalized again at the clinic in connection with shortness of breath and fatigue, progressing from about one week. There were clinical, laboratory, chest X-ray, and CT data for spontaneous bilateral hemorrhagic pleural effusions; the bigger one was on the right side (Figure 4).

**Figure 5:**
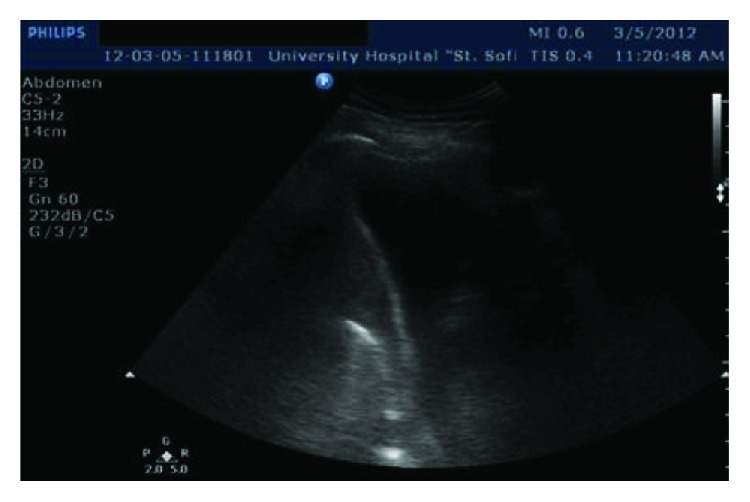
Ultrasound examination of the chest—a pleural effusion with a liquid and a solid (axillary and interlobar) component, evaluated as a hematoma, confirmed in the following surgical revision.

**Figure 6:**
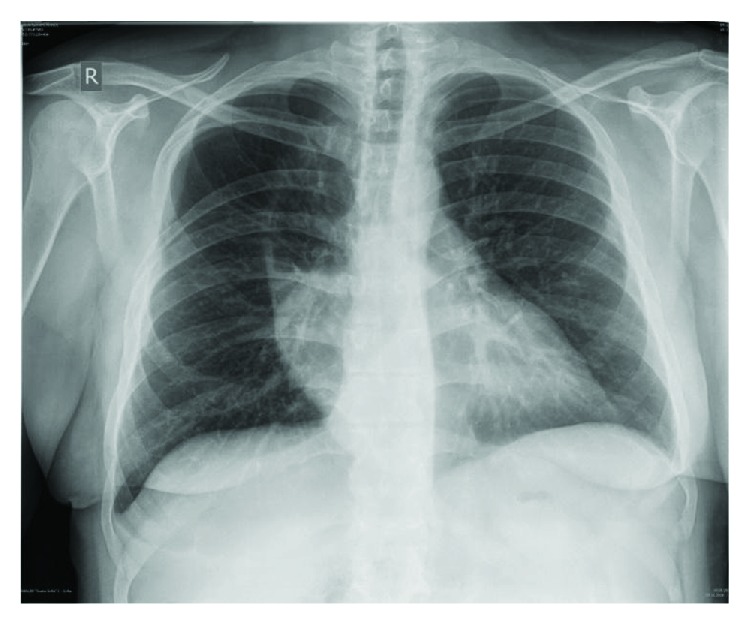
Chest X-ray after the decortication.

Thoracentesis and drainage were performed on the right side with the evacuation of 1200 ml hemorrhagic exudate and on the left side with the evacuation of about 400 ml hemorrhagic fluid. Three days later because of chest X-ray and ultrasound data (Figure 5) for a coagulated right-sided hemothorax, a posterior-lateral right thoracotomy followed. The coagulated hematoma in a stage of organization was evacuated, tightly located over the whole visceral pleura, and there was near 1500 ml of dark liquid hemorrhagic component, placed in many loculations, which was also successfully removed. Debridement, decortication of the right lung, and partial pleurectomy were undertaken. Any source of the hemorrhage was established intraoperatively. The gastric transplant in the posterior mediastinum was found intact. The patient was discharged in a clinically stable general condition after a smooth postoperative period (Figure 6).

A following hospitalization at the TSC came one year later on the occasion of complaints that began a few weeks earlier and consisted of pain and heaviness in the operational scar area in the abdomen, with the clinical evidence of a postoperative herniation of the anterior abdominal wall about 8/8 cm in size.

A further surgical intervention to the patient was undertaken—herniotomy and plastics of the anterior abdominal wall with polypropylene fiber. The patient was released clinically stable after a smooth postoperative period without any registered subsequent postoperative complications.

The follow-up of the patient was documented two years later an anteroseptal myocardial infarction—STEMI with PCI and stent implantation—DES x 1 LADm. Further following of the case in a gastroenterology clinic for the severity of the liver damage to be assessed after discontinuation of the maintenance treatment with Exviera and Viekirax detected no HCV-RNA, which determined the sustained virologic response to treatment.

## 2. Discussion

The Bardet-Biedl syndrome is an extremely rare autosomal recessive disorder with a wide spectrum of multiorgan system clinical manifestations. The first clinical cases were reported by Laurence and Moon in 1866, who described 4 cases in a 10-member family [1]. Georges Bardet and Arthur Biedl established in 1922 the interrelationship between the different symptoms and so included their names in the name of the syndrome [1]. Currently, some researchers do not agree that the syndromes described, respectively, by Laurence-Moon and Bardet-Biedl represent the same genetic disorder [2]. The main characteristics are retinitis pigmentosa with impairment mainly of the night and peripheral vision, nystagmus, polydactyly, marked central type obesity, mental retardation, hypogonadism, and renal dysfunction. Other signs that can be met are hepatic fibrosis, diabetes mellitus, neurological and speech deficiency, progressive weakness leading to paraplegia, behavior disorders, brachycephalia, facial dysmorphism, dental anomalies, and congenital heart block [2, 3]. The full range of features occurs in only 40-45% of cases [4]. There have been cloned totally 21 genes responsible for the development of the disease—BBS1-BBS21 [5]. Their proteins are components of the centrosomes and affect the ciliary transport, which is the cause of why the syndrome belongs to the so-called “ciliopathies” [6]. The incidence of the disease is 1 : 160 000 and in the literature is described as primarily familial cases [7–9]. The frequency is much higher in certain populations with a high level of consanguinity between parents or those with geographical isolation, such as some populations in Newfoundland and Kuwait with affected 1 : 13 000 and 1 : 17 000 live births, respectively [2].

The current clinical case demonstrates a rare genetic syndrome, which progressed to a terminal renal failure and imposed kidney transplantation with a good posttransplant function. Subsequent HCV and CMV infections were detected in the context of immunosuppressive treatment, and both infections were treated successfully, during the follow-up period with no evidence of viremia. Malignant esophageal disease was diagnosed—a moderately differentiated adenocarcinoma at an early stage without the presence of local and distant dissemination. Complicated surgical treatment was performed to achieve tumor control in the condition of a kidney transplant. Several postoperative complications that occurred at a different stage after the initial surgery were successfully resolved. Spontaneous pleural hemorrhage due to immunosuppressive therapy with Cyclosporin A is extremely rare, but it was observed in the current clinical case. At the present time, the patient strictly adheres to the prescribed treatment for all of his diseases and has a satisfactory quality of life.
